# Affinity Purification of Angiotensin Converting Enzyme Inhibitory Peptides from Wakame (Undaria Pinnatifida) Using Immobilized ACE on Magnetic Metal Organic Frameworks

**DOI:** 10.3390/md19030177

**Published:** 2021-03-23

**Authors:** Xuezhen Feng, Dankui Liao, Lixia Sun, Shanguang Wu, Ping Lan, Zefen Wang, Chunzhi Li, Qian Zhou, Yuan Lu, Xiongdiao Lan

**Affiliations:** 1 Guangxi Key Laboratory of Petrochemical Resource Processing and Process Intensification Technology, School of Chemistry and Chemical Engineering, Guangxi University, Nanning 530004, China; fengxuezhenbest@163.com (X.F.); binglin0628@163.com (L.S.); wangzefen@126.com (Z.W.); 1814304019@st.gxu.edu.cn (C.L.); 15532370620@163.com (Q.Z.); 2 Medical College, Guangxi University of Science and Technology, Liuzhou 545006, China; wsg_gxust1974@163.com (S.W.); luyuan0606@163.com (Y.L.); 3 Guangxi Key Laboratory of Polysaccharide Materials and Modifications, School of Chemistry and Chemical Engineering, Guangxi University for Nationalities, Nanning 530008, China; gxlanping@163.com

**Keywords:** magnetic zeolitic imidazolate framework, immobilization, affinity purification, angiotensin converting enzyme inhibitory peptides

## Abstract

Angiotensin-I-converting enzyme (ACE) inhibitory peptides derived from marine organism have shown a blood pressure lowering effect with no side effects. A new affinity medium of Fe_3_O_4_@ZIF-90 immobilized ACE (Fe_3_O_4_@ZIF-90-ACE) was prepared and used in the purification of ACE inhibitory peptides from Wakame (*Undaria pinnatifida*) protein hydrolysate (<5 kDa). The Fe_3_O_4_@ZIF-90 nanoparticles were prepared by a one-pot synthesis and crude ACE extract from pig lung was immobilized onto it, which exhibited excellent stability and reusability. A novel ACE inhibitory peptide, KNFL (inhibitory concentration 50, IC_50_ = 225.87 μM) was identified by affinity purification using Fe_3_O_4_@ZIF-90-ACE combined with reverse phase-high performance liquid chromatography (RP-HPLC) and MALDI-TOF mass spectrometry. Lineweaver–Burk analysis confirmed the non-competitive inhibition pattern of KNFL, and molecular docking showed that it bound at a non-active site of ACE via hydrogen bonds. This demonstrates that affinity purification using Fe_3_O_4_@ZIF-90-ACE is a highly efficient method for separating ACE inhibitory peptides from complex protein mixtures and the purified peptide KNFL could be developed as a functional food ingredients against hypertension.

## 1. Introduction

As a zinc-dependent carboxypeptidase, angiotensin-I-converting enzyme (ACE EC 3.4.15.1) is prolific in biological tissues and blood, and plays an important role in modulating blood pressure [1]. ACE cannot only convert angiotensin I to angiotensin II (a potent vasoconstrictor), but it can also degrade the vasodilator bradykinin [2]. ACE inhibitors are, thus, a class of drugs frequently used in the treatment of hypertension. Synthetic ACE inhibitors, including captopril, benazepril, enalapril, and perindopril are associated with various side effects, such as cough, taste disturbances, allergic reactions, and skin rashes [1]. However, ACE inhibitors derived from food have displayed some potential for reducing blood pressure with fewer adverse effects [3,4]. Thus, the food-derived ACE inhibitors have become popular in hypertension management, making ACE an attractive target for drug discovery [5,6].

Research has shown that the screening of ACE inhibitors was usually carried out by using free ACE during the process of isolation and purification. The purification of ACE from tissues (such as pig lung or cow lung) is not a simple task, and the resultant product loses activity very quickly due to the temperature, pH, and operating force required during the purification of ACE [7,8]. Moreover, commercial ACE is very expensive, and about 100 activity assays can be accomplished with 1 unit [9,10]. Based on this, the immobilization of ACE is an important issue for assay development, as immobilization has the potential to extend its thermal stability and promote enzyme recycling [11,12].To date, some support for immobilized ACE, such as agarose [7], magnetic beads [8], and Zn-SBA-15 [12] have been developed for anchoring ACE. For example, the ACE crude extract from pig lungs was immobilized onto glyoxyl agarose via a Schiff’s base reaction, and it was demonstrated that the immobilized ACE could be used for activity assays and inhibitor screening [7,13]. ACE has also been chemically immobilized on ferrite magnetic beads, and this was used to identify the reference inhibitor (Lisinopril) [8]. Based on the advantages of magnetic materials in the field of separation, Lan et al. reported rapid purification and characterization of ACE inhibitors using magnetic agarose-immobilized ACE [6,11]. As immobilized metal ion affinity media (IMAM), Zn-SBA-15-ACE was used to purify ACE inhibitors from Volutharpa ampullaceal perryi. This previous research suggests that affinity purification is an efficient protein purification method relying on the formation of specific reversible complexes between an immobilized molecule and the ligands to be purified [14].

Some progress has been made in recent years, however, due to the large ACE molecular dimensions (6.2 nm × 7.5 nm ×6.5 nm), coupled with ease of inactivation, and cost, research on the immobilization carriers for ACE is still relatively rare. Therefore, it is of high importance to develop an efficient immobilized support onto ACE for affinity purification of ACE inhibitors. Metal-organic frameworks (MOF) exhibit attractive properties for use as potential supports for enzyme immobilization, such as tunable pore size, optional surface functionalization, and high guest loading efficiency [15,16]. As an important class of MOF, zeolitic imidazolate framework (ZIF) materials exhibit promising properties with their chemical and thermal stability [17]. Moreover, ZIFs can be combined with magnetic nanoparticles (Fe_3_O_4_NPs) to form core-shell nanocomposites, which can be easily recycled and reused by an external magnet [18,19]. Moreover, Zn^2+^ in ZIFs can coordinated with imidazolyl (histidine), thiol sulfur (tryptophan), and indolyl (cysteine) functional groups which was used to isolate selectively proteins or peptides [20]. Among ZIFs, ZIF-90 is an excellent potential candidate for post-synthetic modification, with a free aldehyde group on the imidazole-2-carboxaldehyde (ICA) ligand which can react with amine-containing materials by a Schiff base reaction [21,22,23].The protease trypsin was successfully immobilized onto Fe_3_O_4_@DOTA-ZIF-90, and the immobilized trypsin exhibits satisfactory tryptic digestion efficiency [24]. These showed that magnetic ZIF-90 can not only be a medium of affinity purification, but also an effective carrier for the immobilization of enzymes.

As one of the most abundant edible wild algae species in Asia, Wakame has wide distribution, high production, and it has become a popular food. Yamori et al. reported that the water-soluble fiber of wakame efficiently decreased blood pressure in spontaneously hypertensive rats [25]. To date, there was 21 ACE inhibitory peptides which have been isolated and purified from Wakame by hydrolysis, ion exchange chromatography, gel filtration and RP-HPLC [26,27,28]. To broaden its application as an active ingredient for hypertension treatments, more ACE inhibitory peptides must be obtained from Wakame. Different ACE inhibitory peptides can be obtained from the same hydrolysates by different purification methods [6,29].

**Figure 1:**
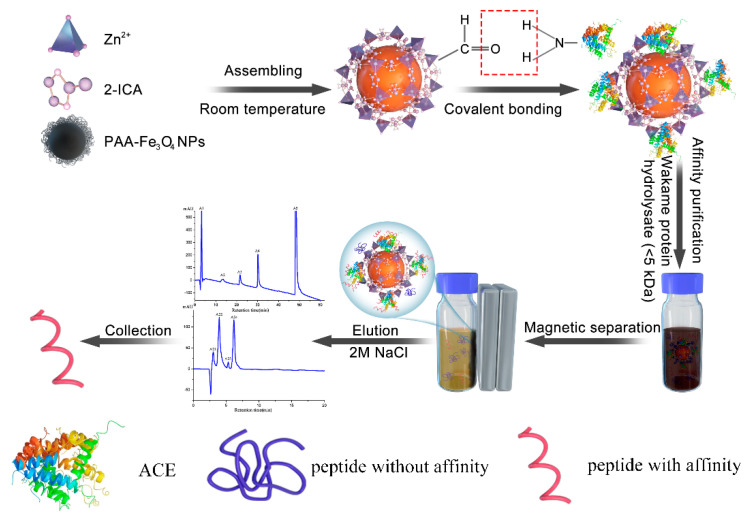
Schematic diagram of synthesis of Fe_3_O_4_@ZIF-90-ACE and affinity purification process. (2-ICA is the abbreviation of Imidazole-2-carboxaldehyde; ACE is the abbreviation of angiotensin-I-converting enzyme).

In our study (Figure 1), we prepared Fe_3_O_4_@ZIF-90 nanoparticles to immobilize crude ACE from pig lungs (Fe_3_O_4_@ZIF-90-ACE), and the immobilization mechanism was studied by XPS and Density Function Theory. Magnetic affinity purification has the advantage of fast separation and ease of recycling. A novel ACE inhibitory peptide was separated from Wakame protein hydrolysate (WPH) (<5 kDa) by affinity purification based on Fe_3_O_4_@ZIF-90-ACE. The inhibitory activities and inhibition pattern were also confirmed by synthetic samples. Moreover, the interaction mechanisms of the inhibitory peptides with N-ACE and C-ACE were carried out using AutoDock Vina.

## 2. Results

### 2.1. Characterization of Fe_3_O_4_@ZIF-90 and Immobilized ACE

**Figure 2:**
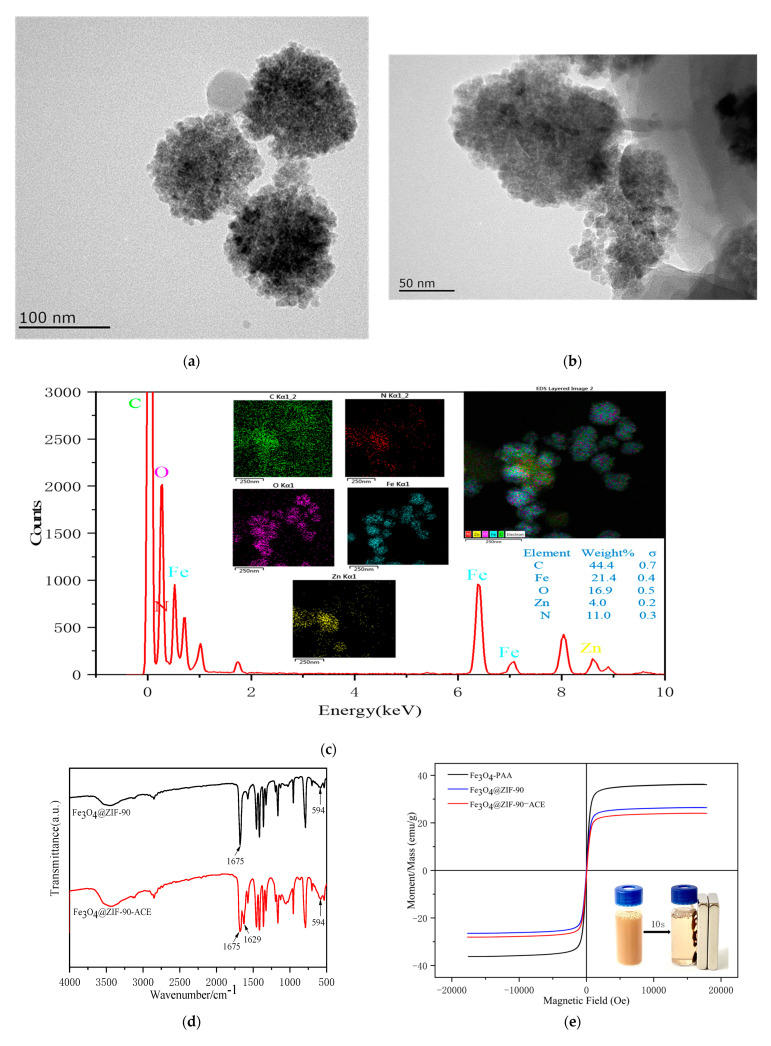
(**a**) TEM image of Fe_3_O_4_@ZIF-90 (**b**)TEM image of Fe_3_O_4_@ZIF-90-ACE, (**c**) EDS of Fe_3_O_4_@ZIF-90, (**d**) FT-IR spectrum, (**e**) the field-dependent magnetization curves of the materials (insert of separation of Fe_3_O_4_@ZIF-90-ACE from solution within 10s using a magnet).

The morphologies of the products were observed by TEM. Figure 2a explicitly shows that the Fe_3_O_4_@ZIF-90 particles were spherical and uniform, with an average particle size of approximately 100–130 nm. The outside surface texture of Fe_3_O_4_@ZIF-90 was coarse and a core-shell structure was also observed, which was consistent with the reports of Nosike et al. [23].There were slight changes observed in the morphology after linking ACE to the surface of the nanocomposite, as seen in Figure 2b. In addition, Figure 2c shows the EDS spectra and elemental mapping for Fe_3_O_4_@ZIF-90, which further establishes the core-shell structure according to the appearance of N and Zn [23]. As shown in Figure 2d, a characteristic band for the −CHO stretching mode was observed at 1675 cm^−1^ in Fe_3_O_4_@ZIF-90 [23]. The adsorption peak at 1629 cm^−1^ corresponds to the stretching vibration of C=N, which represents the amide bond arising from ACE immobilized on the surface of Fe_3_O_4_@ZIF-90 by the Schiff reaction [24].

The hysteresis loops passed through the original point (Figure 2e), and the materials exhibited super-paramagnetism. The saturation magnetization (Ms) value for PAA-Fe_3_O_4_NPs, Fe_3_O_4_@ZIF-90 and Fe_3_O_4_@ZIF-90-ACE were 36.12, 26.41, and 24.00 emu·g^−1^ at 300 K, respectively. As shown in Figure 2e (inset), Fe_3_O_4_@ZIF-90-ACE can be separated from solution within 10 s using a magnet.

### 2.2. Stability of Fe_3_O_4_@ZIF-90 Immobilized ACE

**Figure 3:**
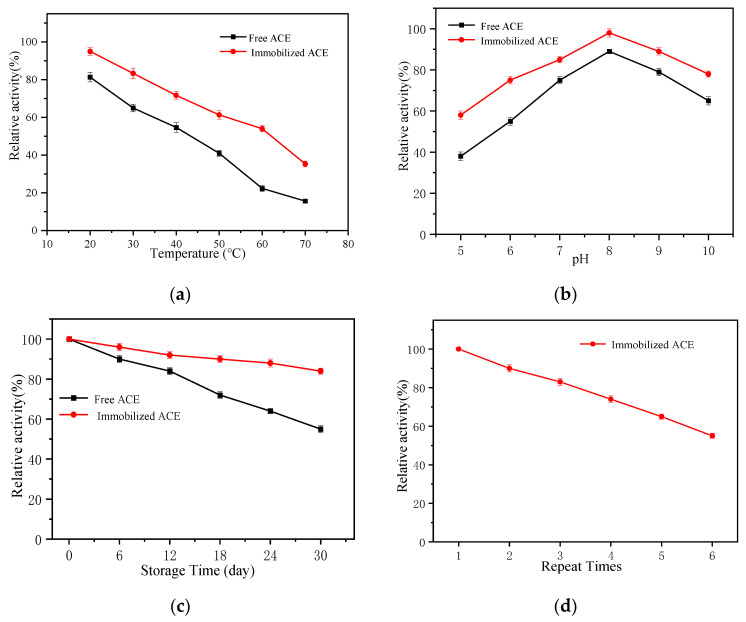
Temperature stability (**a**); pH stability (**b**); storage stability (**c**); and operational stability; (**d**) of free and immobilized ACE.

To evaluate the thermal, pH and storage stabilities and reusability of the immobilized ACE, the residual activities were measured and the results are shown in Figure 3. The immobilized ACE retained 54.49% activity and the free ACE only had 22.03% activity (Figure 3a) after heating at 60 °C for 1 h, showing that the immobilized ACE was more thermally stable relative to the free ACE. This may be due to an increase of ACE rigidity by immobilization preventing conformation variation at high temperatures [30]. As seen from Figure 3b, the immobilized ACE retained more than 58.39% relative activity at pH 5.0–10.0, while the activity maintenance of the free enzyme was only 37.05%. This suggests that the immobilized ACE has better pH stability compared with the free ACE.

Storage stability of ACE exhibited a significant improvement after immobilization (Figure 3c). The free enzyme lost 45.04% of its initial activity, whereas the immobilized ACE preserved about 84.09% of its original activity after 30 days under the same storage conditions. The reusability of immobilized ACE was investigated over six cycles (Figure 3d). The relative activities decreased as the cycle number increased, but the immobilized ACE achieved an activity retention rate of 54.05% after the sixth cycle. These results suggest that the strategy to immobilize the enzyme through chemical crosslinking may help to preserve the enzymatic activity and retain excellent operational stability. We attribute the above results to the variations in the conformation of the enzyme upon covalent bond formation and changes in microenvironment upon immobilization.

### 2.3. The Adsorption Mechanism of Fe_3_O_4_@ZIF-90-Immobilized ACE

**Figure 4:**
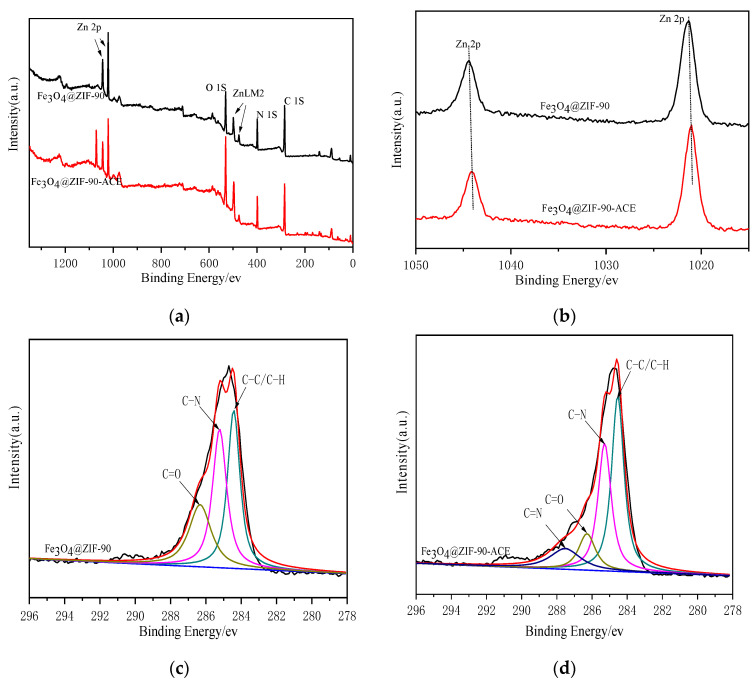
(**a**) The XPS spectra of full survey; (**b**) Zn2p core level spectra of Fe_3_O_4_@ZIF-90 and Fe_3_O_4_@ZIF-90-ACE; C1s spectra of Fe_3_O_4_@ZIF-90; (**c**,**d**) Fe_3_O_4_@ZIF-90-ACE.

XPS analysis of Fe_3_O_4_@ZIF-90 and Fe_3_O_4_@ZIF-90-ACE was carried out to further explore the mechanism of adsorption. As shown in Figure 4a, peaks corresponding to C, N, O, Zn, and Fe were present in the XPS spectra of the Fe_3_O_4_@ZIF-90 and Fe_3_O_4_@ZIF-90-ACE [31]. The XPS peaks at a binding energy of Zn 2p1/2(1021.3 eV) and Zn 2p3/2(1044.4) eV observed for Fe_3_O_4_@ZIF-90 confirmed the existence of ZIF-90 frameworks (Figure 4b), however, there were slight changes observed in the binding energy of Zn 2p1/2 (1021.1 eV) and Zn 2p3/2 (1044.3) eV in Fe_3_O_4_@ZIF-90-ACE [32,33]. This indicated that Zn (II) had complexed with functional groups in ACE during the adsorption process [34]. This may be occurring via the external active site Zn^2+^ (Zn-OH), obtained by the dissociation of water on the ZIF-90 surface, which can coordinated with imidazolyl (histidine), thiol sulfur (tryptophan), and indolyl (cysteine) functional groups in ACE. Based on this, the adsorptive separation of ACE from pig lung has been demonstrated [6,11]. The C1s peaks of Fe_3_O_4_@ZIF-90 and Fe_3_O_4_@ZIF-90-ACE are shown in Figure 4c,d, respectively. The binding energies of C-containing groups changed before and after adsorption. The lower binding energies of C=O may be caused by a hydrogen bond formation process during the immobilization. A new peak, which was attributed to the C=N bond generated after the immobilization, indicating that the ACE was not only physically adsorbed on the support, but was also bonded to the ZIF-90 skeleton chemically [34,35]. This phenomenon suggested that during the adsorption process, the electrophilic carbon atoms of aldehyde groups of the ZIF-90 were nucleophilically attacked by the amine group of the ACE to form imine linkages, also known as Schiff base linkages.

Covalent coupling based upon Schiff base is one of the most common immobilization methods. The immobilized crude ACE from pig lung is not pure, and adsorbent ZIF-90 has good adsorption capacity, so, using ZIF-90 as an example, the modeling of the binding sites of key amino acid residues in ACE was built by a density functional theory (DFT) in geometric optimization [36,37].

**Figure 5:**
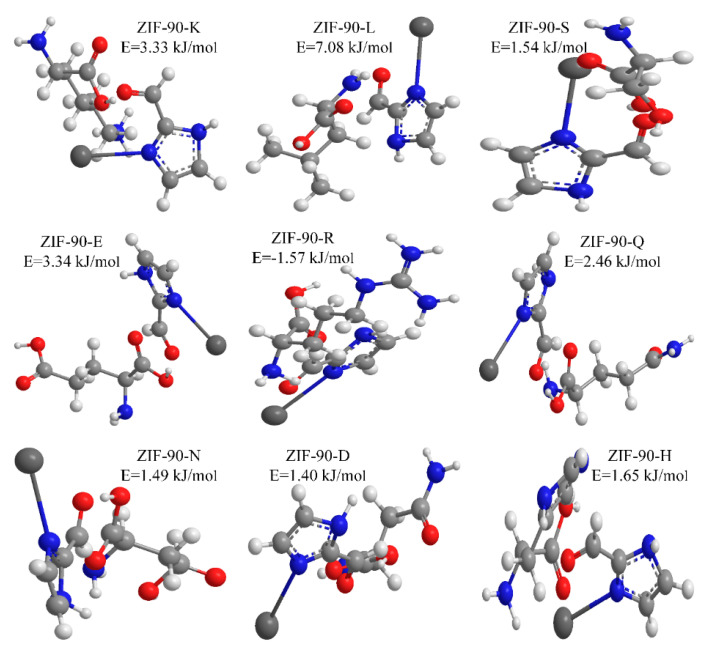
The Binding state of ZIF-90 to amino acids (blue for N, red for O, light gray for C, dark gray for Zn; K for Lys, L for Leu, S for Ser, E for Glu, R for Arg, Q for Gln, N for Asn, D for Asp, H for His; E stands for the adsorption energy.).

The experiment results of XPS showed that amino acids (such as L,K,R) were bonded to the ZIF-90 skeleton chemically by Schiff base reaction. Furthermore, the hydrophilicity amino acids are placed in the external regions of the enzyme molecule and, so, are theoretically available to take part in reactions of cross-linking and bonding with surfaces without the need for structural changes in the molecule’s active center responsible for biological activity. So, the key amino acids used for calculations mainly involved two sorts: covalent bonding amino acids (L,K,R) and strong hydrophilicity amino acids (S,E,Q, et al.). The interaction between ACE and ZIF-90 was investigated for two possible paths: (1) creation of covalent bonds and (2) physical adsorption between ACE via external amino acids with the support ZIF-90. Based on these, we choose nine key amino acids in ACE to discuss the binding sites on ZIF-90 by a DFT in geometric optimization. Moreover, a deeper understanding of the interaction mechanism between ACE and ZIF-90 can be obtained. The optimized geometries of the complexes and the lowest adsorption energy are illustrated in Figure 5. Calculations showed that nine amino acids (lysine, leucine, serine, glutamic acid, arginine, glutamine, asparagine, aspartic acid, and histidine) can be adsorbed efficiently on the ZIF-90. Among these, leucine had a weak affinity to ZIF-90, whereas aspartic acid had the strongest affinity, with stronger affinity indicating that the adsorption was more likely to occur [34]. In the ACE molecule, hydrogen bonding sites (e.g., C=O and C-N) and hydrophilic groups (e.g., −CHO) of ZIF-90 were highly distributed, and intermolecular hydrogen bonds could be formed between the ZIF-90 and the amino acids. The intermolecular hydrogen bonding was also the main source of the mutual attraction between the carrier and the enzyme molecule [38]. Factors such as solvent, temperature, concentration, and pH can affect the formation of hydrogen bonds. This deduction was evidenced by the different amounts of immobilization under different conditions (Figure S4). The results from XPS and DFT suggest that the immobilization process not only involves covalent bonding, but also physical adsorption.

### 2.4. Purification and Identification of ACE Inhibitor Peptides from Wakame Protein Hydrolysate

**Figure 6:**
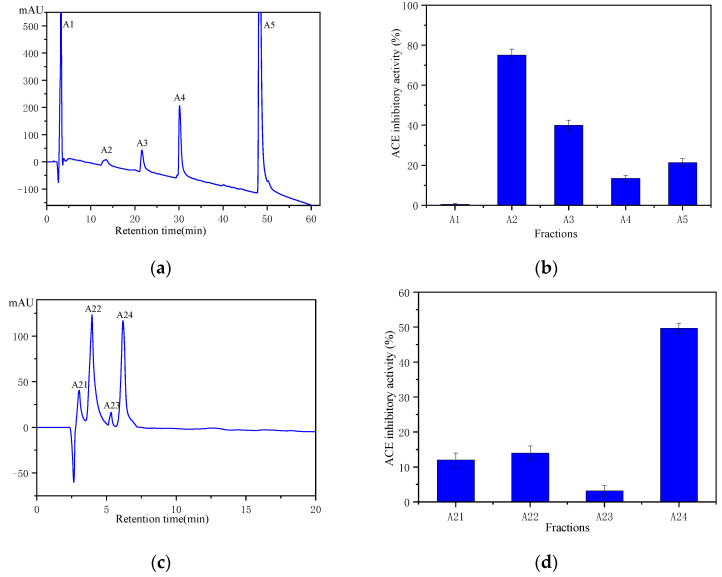
Chromatographic purification on a Zorbax SB C18 column of ACE inhibitory peptides obtained by Fe_3_O_4_@ZIF-90-ACE affinity medium and ACE inhibitory activity evaluation. (**a**) Separation was performed with a linear gradient of acetonitrile in water containing 0.1% TFA (0–100% in 60 min) at a flow rate of 1 mL/min. (**b**) ACE inhibitory activity of fractions A1 to A5 were assayed with a concentration of 0.42 mg/mL. (**c**) Separation of fraction A2 was performed with a linear gradient of acetonitrile in water 19 (containing 0.1% TFA) from 15% to 50% in 20 min at a flow rate of 0.5 mL/min. (**d**) ACE inhibitory activity of fractions A21 to A24 were assayed with a concentration of 0.12 mg/mL.

**Figure 7:**
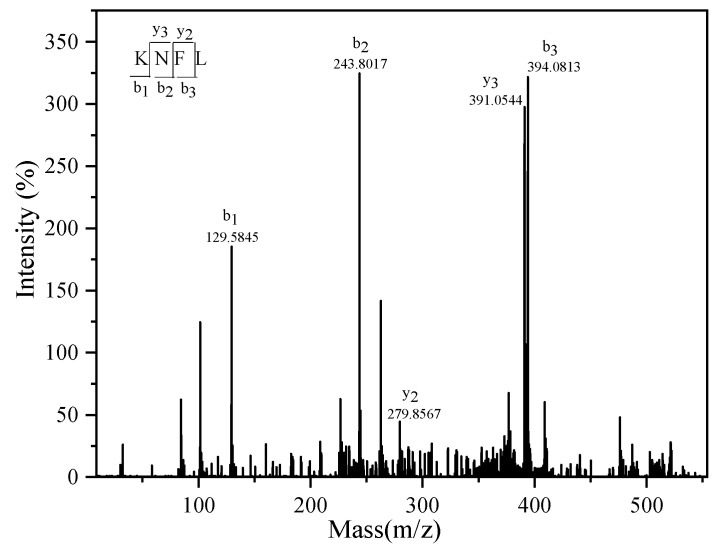
Peptide profile of A24 fraction performed by mass spectrometry analysis.

Fe_3_O_4_@ZIF-90-ACE was used for affinity purification of ACE inhibitor peptides from Wakame protein hydrolysate (WPH). The WPH (<5 kDa) was fractionated by affinity purification and a two-step RP-HPLC as shown in Figure 6. The eluted, bound peptides were purified into five fractions by RP-HPLC, in which the A2 fraction showed the highest ACE inhibitor activity (Figure 6a,b). In Figure 6a, fractions A1 had less ACE inhibitory activity, suggesting that there was nonspecific adsorption to the affinity medium Fe_3_O_4_@ZIF-90-ACE. The reason could be the immobilized crude ACE from pig lung might bind not only to ACEI, but also non-inhibitor peptides for ACE on account of other immobilized molecules on Fe_3_O_4_@ZIF-90 [6]. Fraction A2 was further separated using RP-HPLC to provide four fractions (Figure 6c,d). Fraction A24 exhibited the highest ACE inhibitor activity, with an inhibitory concentration 50 (IC_50_) value of 0.12 mg/mL. Fraction A24 was identified through MALDI-TOF–TOF mass spectrometry (Figure 7), and its molecular mass was determined to be 524.1 Da. On the basis of this molecular mass and tandem MS, the amino acid sequence was identified as Lys-Asn-Phe-Leu (KNFL). The IC_50_ value of A24 was 228.96 ± 1.5 μM, while the IC_50_ value of the chemically synthesized peptide (98% purity) was very similar, at 225.87 ± 2.7 μM. No matching amino acid sequence was found when the database was searched (AHTPDB, https://webs.iiitd.edu.in/raghava/ahtpdb/source_browse.php?name=fish (accessed on 1 September 2020), BIOPEP http://www.uwm.edu.pl/biochemia/biopep/start_biopep.php (accessed on 1 September 2020), and EROP—Moscow http://erop.inbi.ras.ru/ (accessed on 1 September 2020)) for retrieval. Herein, a novel ACE inhibitory peptide, KNFL, was rapidly purified from Wakame for the first time using affinity medium Fe_3_O_4_@ZIF-90-ACE. 

Compared with conventional separation technologies, such as ion exchange chromatography and gel filtration chromatography, affinity purification for the preparation of ACE inhibitory peptides has attracted widespread attention due to its simplified process and less time [6,12,39]. The medium on affinity purification of the ACE inhibitory peptides mainly includes the immobilized ACE and immobilized metal affinity medium (IMAM) (Table 1). As seen from Table 1, different ACE inhibitory peptides derived from Lizard fish were obtained by the conventional approach, affinity purification using IMAC-Ni^2+^ and magnetic agarose-ACE [6,29,39]. Moreover, the similar phenomena have also occurred on casein and wakame. Twenty-one ACE inhibitory peptides were purified from Wakame, including 17 dipeptides and 4 tetrapeptides by hydrolysis, ion exchange chromatography, gel filtration and RP-HPLC [26,27,28]. Among the four tetrapeptides, the highest IC_50_ value was 213 μM, which is lower than that of KNFL (225.87 ± 2.70 μM) in our study. The reason may be that a few of changes have taken place on the structure of immobilized ACE (Figure S5c,d), affecting coordination with the peptide and the IC_50_ was determined by free ACE, which also may be the reason for the higher IC_50_ (2.08 mM/4.66 mM) of the ACE inhibitory peptide by the affinity purification using Zn-SBA-15-ACE [12]. However, structural change of immobilized ACE and coordination with the active peptide may be comparatively complex, the reverse applied in the Lizard fish (Table 1). These indicate that different ACE inhibitory peptides could be obtained by affinity purification method and conventional approach, and enriched based on different specific affinity. Therefore, the affinity purification is an effective method to obtain novel ACE inhibitory peptides. In terms of IMAM, the preparation process of IMAM is relatively complicated. The magnetic IMAM (IMAM @ mPEG) was prepared by the synthesis of magnetic silica (mSiO_2_), amination modification, epichlorohydrin activation, the composition of IMAM (mSiO_2_@Cu^2+^) and aldehyde modification [9,10]. However, Fe_3_O_4_@ZIF-90 could be synthesized in one pot at room temperature which has the advantage of magnetic properties and affinity purification of metal ions. Moreover, the preparation of Fe_3_O_4_@ZIF-90-ACE was easily obtained by shaking without additional activation [6]. In addition, immobilization of crude ACE could not only save cost, but could also improve stability and operability. In conclusion, purifying bioactive peptides using Fe_3_O_4_@ZIF-90-ACE is an efficient approach and some novel ACE inhibitory peptides may be gained from the same source.

Researchers have investigated the structure-activity relationship of ACE inhibitor peptides, such as the peptide chain length, amino acid sequence, C- terminal, and N-terminal amino acid sequence [4,42]. Most of the ACE inhibitors are reported to have small molecular weights (2–12 amino acids), and there are four amino acids in the newly identified peptide KNFL. Hydrophobic amino acids at the C-terminus of ACE inhibitor peptides have been shown to provide an enhanced inhibitor effect [43]. In this study, the C-terminal amino acids (phenylalanine and leucine) of the KNFL peptide have strong hydrophobicity, which confers potency; an effect further supported by ACE inhibitor peptides FFL, IFL, and AFL in Table 2. Furthermore, previous studies have shown that the presence of aromatic or alkaline amino acids at the N-terminus are beneficial for higher inhibitor activity [44]. The lysine at the N-terminus of KNFL is an alkaline amino acid, which promotes inhibitor activity, such as with KNGDGY in Table 2. For example, the ACE inhibitor peptide KW has an IC_50_ of 7.8 μM [45]. Whereas that of LW is 50 μM [46]. These factors also helped to enhance the inhibitory effect of the isolated ACE inhibitor peptide.

### 2.5. Inhibition Pattern of ACE Inhibitor Peptide KNFL

**Figure 8:**
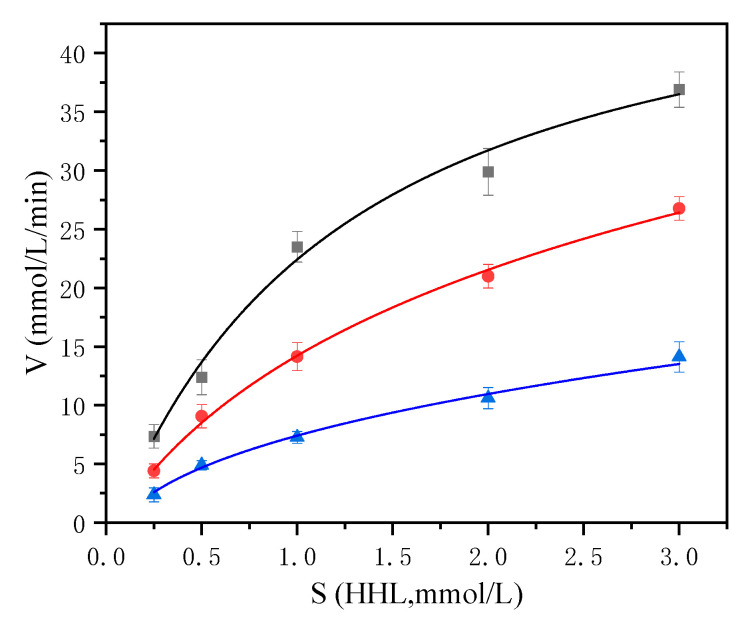
The nonlinear fitting plots of the ACE inhibitory pattern of the active peptide KNFL.

ACE inhibitor peptides have been reported with competitive, noncompetitive, uncompetitive, and mixed modes of interaction. The kinetic constants were estimated by using nonlinear regression analysis (SPSS, version 26.0, Inc., Chicago, IL, USA) and the nonlinear fitting plots were shown in Figure 8. The kinetic constants of ACE in the presence of KNFL revealed that the maximum reaction velocity (*V*_max_) values decreased in a dose-dependent pattern, while the *K*_m_ values increased, which demonstrates mixed-type inhibition (Table 3). Mixed-type inhibitors interact with the non-catalytic site of the enzyme and combine with the enzyme to produce an ACE–HHL(Hip-His-Leu) peptide complex which might leads to conformational changes and a decrease in substrate affinity at the active site resulting in loss of activity [50]. A great many of ACE inhibitor peptides acted in a mixed-type manner. For example, the MAINPSKENLCSTFCK peptide produced from casein hydrolysates [50], and EVSQGRP, VSRHFASYAN and SAAVGSP generated from *Stichopus horrens* [51].

### 2.6. Molecular Interaction between ACE Inhibitors Peptides and ACE

**Figure 9:**
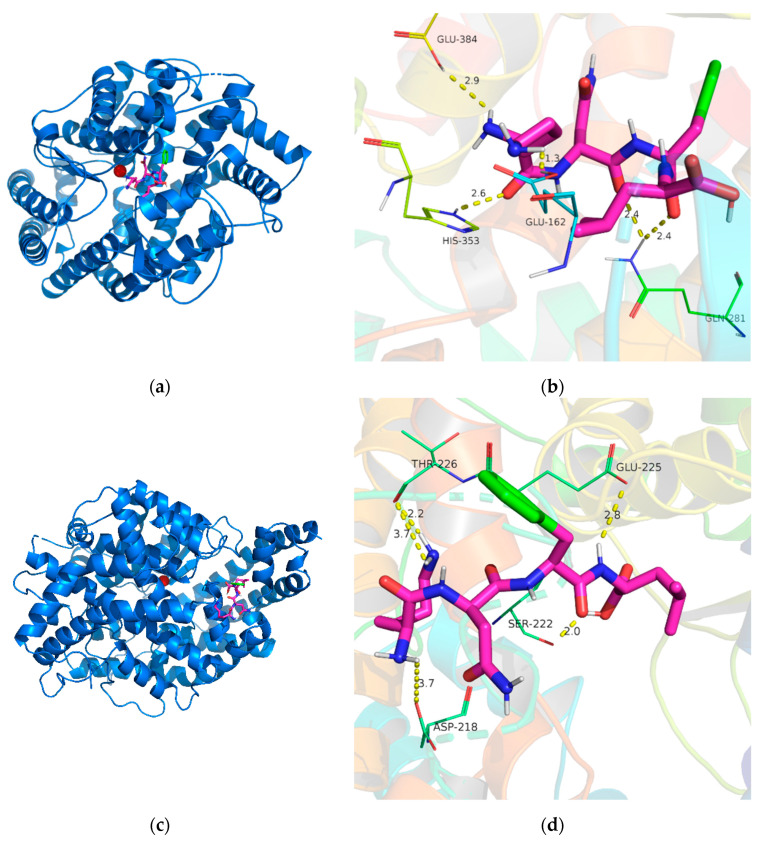
The molecular docking simulation of KNFL binding to ACE. (**a**) The docking simulation of KNFL (green) binding to active sites of ACE (shown as cartoon). A zinc ion (red) was present in the active site of ACE. (**b**) The interaction between KNFL (shown as sticks) and the residues of ACE (shown as lines) is shown. Yellow dash indicates H bonding. (**c**) The docking simulation of KNFL (green) binding to non-active sites of ACE (shown as cartoon). A zinc ion (red) was present in the active site of ACE. (**d**) The interaction between KNFL (shown as sticks) and the residues of ACE (shown as lines) is shown. Yellow dash indicates H bonding.

Molecular docking studies of the inhibitory peptide KNFL with ACE were carried out using AutoDock Vina to evaluate the interaction mechanism. The mixed-type inhibition indicates that the peptide binds to the ACE at positions including both the active and non-active sites, which consequently reduces the catalytic activity of ACE. According to the results of the inhibition pattern, the docking was performed on both the active and non-active sites; the results are shown in Figure 9.

As shown in Figure 9a,b, KNFL bound to active site of ACE (Figure 9a), and five hydrogen bonds with residues Glu162 (1.3 Å), Gln 281(2.4 Å, 2.4 Å), His353 (2.6 Å), and Glu384 (2.9 Å) were generated (Figure 9b). The shorter the length of a hydrogen bond, the greater the binding forces, so Glu formed the strongest hydrogen bond [52]. Hydrogen bonds are the main interaction forces which stabilize the structure of non-catalytic enzyme−peptide complexes [53]. The KNFL peptide also formed five hydrogen bonds with the non-active sites of ACE. The involved residues were Ser222, Thr226, Glu225, and Asp218 (Figure 9d). The Lys of the peptide forms two H bonds with Thr226 (2.2 Å and 3.7 Å), Asn forms one H bond with Ser222 (2.0 Å), Phe forms one H bond with Glu225 (2.8 Å), and Leu forms one H bond with Asp218 (3.7 Å). 

Moreover, the binding affinity of KNFL interacting with the active and non-active site of ACE were −6.9 kcal/mol and −5.6 kcal/mol, respectively, indicating that the KNFL peptide could bind with the active and non-active site of ACE via hydrogen bonds.

## 3. Materials and Methods

### 3.1. Materials and Chemicals

Wakame sample was collected from the Beibu Gulf of Guangxi (20°54′10″−21°40′30″ N, 109°05′20″−109°11′35″ E) and was identified by Dr. Kun Xing of Dalian Ocean University as *Undaria pinnatifida*.

Angiotensin I-converting enzyme (ACE, EC3.4.15.1) from rabbit lung, HHL (Hippuryl-l-histidyl-l-leucine), were obtained from Sigma-Aldrich Co. (St. Louis, MO, USA). The ACE inhibitor KNFL (Lys-Asn-Phe-Leu) was supplied by GL Biochem Ltd. (Shanghai, China). Iron chloride hexahydrate (FeCl_3_·6H_2_O), ethylene glycol (EG), diethylene glycol (DEG), sodium acetate anhydrous (NaAc), and zinc acetate dehydrate Zn(CH_3_COO)_2_·2H_2_O were all obtained from Sinopharm Chemical Reagent Co., Ltd. (Shanghai, China). Polyacrylic acid (PAA), imidazolate-2-carboxyaldehyde (2-ICA, 98 %), sodium acrylate (NaAA) were purchased from Macklin Biochemical Co., Ltd (Shanghai, China). The other chemicals and reagents used were of AR grade and used without further purification. 

### 3.2. Synthesis of Fe_3_O_4_@ZIF-90 

The Fe_3_O_4_@ZIF-90 particles were prepared following a reported procedure with some modifications [22]. First, 0.481 g of ICA (0.2 M) was dissolved 25 mL of deionized water, and the solution was added into 1 mg/mL of freshly prepared PAA-modified Fe_3_O_4_NPs under ultrasonic irradiation for 5 min. Then, 25 mL of Zn(CH_3_COO)_2_·2H_2_O aqueous solution (0.1 M) was added, and the mixture was stirred for 10 min at room temperature. The Fe_3_O_4_@ZIF-90 nanocomposites were recovered using an external magnet, washed three times with methanol, and dried in a vacuum oven at 50 °C for 24 h. 

### 3.3. Characterization of ACE Immobilized onto Fe_3_O_4_@ZIF-90

#### 3.3.1. Immobilization of ACE

The ACE was prepared from pig lung and the precipitates were dialyzed and used as a crude enzyme sample for the immobilization of ACE [6]. Twenty-five milligrams of Fe_3_O_4_@ZIF-90 nanoparticles were dispersed in 2 mL of crude sample, and incubated with shaking for a certain time at different temperatures to immobilize the ACE. The immobilized ACE (Fe_3_O_4_@ZIF-90-ACE) was magnetically recovered and the precipitate was washed several times by 0.1 M phosphate (PBS) buffer until the supernatant was free of protein. Immobilized ACE was quantified using the Bradford method, which used bovine serum albumin (BSA) as the standard [54]. Finally, the obtained immobilized ACE was stored at −80 °C. Enzyme loading capacity was calculated using Equation (1):

(1) $$\mathrm{Enzyme}\;\mathrm{loading}\;\mathrm{capacity}(\mathrm{mg}\cdot g^{-1})=\frac{(\mathrm{Total}\;\mathrm{protein}\;\mathrm{content}-\mathrm{supernatant}\;\mathrm{protein}\;\mathrm{content})\;}{\mathrm{Total}\;\mathrm{mass}\;\mathrm{of}\;{\mathrm{Fe}}_{3}O_{4}\text{@}\mathrm{ZIF}-90}$$

#### 3.3.2. The Enzymatic Stability of Immobilized ACE

To determine thermal stability, the immobilized ACE was dispersed in PBS buffer in the absence of the HHL substrate at 20–60 °C for 1 h [55]. The pH stability of immobilized ACE were measured at pH 5.0–9.0 for 1 h. 

The storage stability of immobilized ACE was determined by storing at −20 °C for 30 days. The enzyme activity was then measured, and the relative activity was compared with that of the untreated ACE. The immobilized ACE was evaluated over six successive cycles to investigate the reusability stability; the first-time detection activity was considered as 100%. 

#### 3.3.3. Characterization of Fe_3_O_4_@ZIF-90 and Immobilized ACE

Transmission electron microscopy (TEM) images were taken on a JEM-2100 electron microscope (JEOL, Tokyo, Japan). The elemental composition was determined by X-ray photoelectron spectroscopy (ESCALAB 250Xi XPS, Thermo, MA, USA). Fourier transform infrared spectra were recorded on a Nicolet 6700 spectrophotometer using the KBr disk method (Brook Technology Co., Ltd., New York, MA, USA). Magnetic characterization was carried out on a vibrating sample magnetometer (VSM) 7400 (Lake Shore, Columbus, OH, USA).

#### 3.3.4. The Immobilization Mechanism of Fe_3_O_4_@ZIF-90-ACE

To explore the immobilization mechanism of Fe_3_O_4_@ZIF-90-ACE, the theoretical calculations on the key amino acid residues and ZIF-90 model were performed using the Gaussian 16 software package. The key amino acid residues in ACE are listed in Table 4. The geometry and energy of the amino acid residues and ZIF-90 model were optimized by a density functional theory (DFT) method with M06-2X and 6-31G* level [34,36]. The vibrational frequencies were also calculated until there were no imaginary frequencies to ensure the stability of the optimal structure. The adsorption energy was corrected for basis set superposition error (BSSE) to remove basis function overlap effects and was calculated using Equation (2) [36]:

(2) $${E\;=\;E}_{com}-{(E}_{sub}{\;+\;E}_{ads}{)+\;E}_{BSSE}$$

where *E_com_*, *E_ads_* and *E_sub_* stand for the total energies of complexes, adsorbent and substrate, respectively, *E_BSSE_* represents the BSSE energy.

### 3.4. Purification of ACE Inhibitors Peptides from Wakame

#### 3.4.1. Preparation of Wakame Protein Hydrolysate 

The WPH were prepared according to previous research with some modifications [56]. The Wakame powder (200 g) was dissolved in 0.01 M PBS buffer (pH 7.0) at ratio of 1/20 (*w/v*, g/mL), the mixtures were centrifuged (11,000× *g*, 15 min) to get the supernatant after multi-gelation. The Wakame protein was obtained by ammonium sulfate precipitation (50%) and dialyzed (3.5 kDa MWCO), and then the dialyzed retentate was lyophilized. The freeze-dried Wakame protein (1.0 g) was dissolved in ultrapure water with a ratio of 1:200 (*w/v*) and pretreated by ultrasound at 200 W for 15 min. Then, the bromelain was added at a bromelain/protein molar ratio of 12,000 U/g for 4 h at 45 °C and pH 6.0. Enzymatic hydrolysis was stopped by heating at 90 °C for 15 min. The supernatant was collected by centrifugation (7200× *g*, 15 min) and the enzymatic hydrolysate (WPH) was obtained by ultrafiltration (5 kDa) by Labscale TFF System (Millipore Co., Billerica, MA, USA) for further use. 

#### 3.4.2. Magnetic Affinity Purification of ACE Inhibitory Peptide from WPH

WPH (<5 kDa) (Figure S6) was separated using Fe_3_O_4_@ZIF-90-ACE for purification [6,9]. As an adsorbent, 50 mg of Fe_3_O_4_@ZIF-90-ACE was mixed with WPH (<5 kDa) (2 mL) and stirred at 35 °C for 40 min. The extract was recovered using a magnet and washed several times by PBS until the absorbance of the rinsed buffer at 280 nm reached baseline. The adsorbed peptides were eluted with 2 M NaCl at 30 °C for 30 min an then separated using an RP-HPLC column (Zorbax SB-C18, 4.6 mm × 250 mm, Agilent, Santa Clara, CA, USA) into different fractions. A linear gradient of acetonitrile in water containing 0.1% trifluoroacetic acid (TFA) (5–100% over 60 min) at a flow rate of 1.0 mL/min at 220 nm using a diode array detector (DAD). The fractions collected were concentrated for a further assay of ACE inhibitory activity. The fraction with the highest ACE inhibitory activity was further purified for RP-HPLC using a linear gradient of acetonitrile in water containing 0.1% TFA (15–50% over 30 min) at a flow rate of 0.5 mL/min at 220 nm.

#### 3.4.3. Characterization of ACE Inhibitory Peptide from WPH

The structure of the purified ACE inhibitor peptide was identified using a 4800 plus MALDI TOF/TOF™ Analyzer (Applied Biosystems, Beverly, MA, USA). The sample was mixed with a matrix solution (α-cyano-4-hydroxycinnamic acid solution) and prepared with 50% acetonitrile containing 0.1% TFA [57]. Tandem mass spectrometry (MS) experiments were conducted by collision-induced dissociation, and the peptide sequencing was obtained via tandem MS analysis.

### 3.5. Assay of ACE Activity and Inhibitory Activity

ACE activity was evaluated by measuring the amount of hippuric acid (HA) [6]. The assay mixture (0.3 mL) consisted of 130 µL BBS buffer pH 8.3 (0.1 mM, containing 0.3 M NaCl), 40 µL HHL (5 mM), and different amounts of the free and immobilized ACE. Incubation was carried out at 37 °C for 10 min and was terminated by addition of HCl (0.1 mL, 1 M). The HA content of the mixture was determined by RP-HPLC (Agilent 1260) at 228 nm with a DAD detector. One unit of ACE enzyme activity (U) was defined as the amount of enzyme catalyzing HHL to generate 1 µM of HA per minute under the experimental conditions. 

The ACE inhibitory activity were measured as described above, with the sample replacing the BBS buffer. The ACE inhibition percentage was calculated using Equation (3) [10]:

(3) $$\mathrm{ACE}\;\mathrm{inhibition}\;\mathrm{rate}\%=\frac{\left(A1-A2\right)}{A1}\times 100\%$$

where *A_1_* and *A_2_* are the peak areas of HA in the control group and with inhibitor, respectively.

### 3.6. Molecular Docking

Molecular docking of the ACE inhibitor peptides with ACE was performed by using AutoDock Vina software [49,58]. The crystal structures of ACE (C-sACE, PDB ID: 1O8A) were downloaded from the Protein Data Bank (PDB) (https://www.rcsb.org (accessed on 1 December 2020)). Docking runs were performed using the coordinates of the center position of the site sphere (Active site of ACE: x, 40.556; y, 37.386; z, 43.472) and (non-active site of ACE: x, 41.862; y, 66.265; z, 47.25), respectively. The docking results were visualized using PyMOL1.7.6 to analyze the protein—ligand interaction.

### 3.7. Statistical Analysis

The statistical analysis was performed by using *t*-tests and ANOVA one-way analysis by using SPSS (version 26.0, Inc., Chicago, IL, USA). *p* < 0.05 was taken as statistically significant.

## 4. Conclusions

In summary, this is the first investigation of a new affinity medium of Fe_3_O_4_@ZIF-90-immobilized ACE (Fe_3_O_4_@ZIF-90-ACE), which exhibited excellent stability and reusability. Moreover, the nanocomposite Fe_3_O_4_@ZIF-90 does not need further modification or activation, and the immobilization process can be achieved in one step in solution. Furthermore, a novel ACE inhibitory peptide, KNFL, from WPH was purified by affinity purification using Fe_3_O_4_@ZIF-90-ACE for the first time. This work has demonstrated an efficient affinity purification method for ACE inhibitors from complex protein mixtures, and the purified peptide KNFL may have potential applications in functional foods or as drugs for hypertension treatments. More experimental studies on the antihypertensive activity of KNFL in vivo are in progress and will be reported in due course.

## Figures and Tables

**Table 1:** The summary of ACE inhibitory peptides by conventional approach and affinity purification on different medium.

Sources	Amino Sequence	IC_50_	Method of Purification	Reference
lizard fish	RVCLP	175 µM	Conventional approach	[29]
	RYRP	52 µM	Affinity purification/ IMAC-Ni^2+^	[39]
	GMKCAF	45.7 ± 1.1 µM	Affinity purification/ Magnetic agarose-ACE	[6]
casein	MKP	0.3 μM	Conventional approach	[40]
	WYLHYA	16.2 μM	Affinity purification/ IMAC-Ni^2+^	[41]
	LLYQEPVLGPVR	274 ± 5 μM	Affinity purification/ IMAM @ mPEG	[9]
Pinctada fucata martensii	HLHT/GWA	458.06 ± 3.24 μM/ 109.25 ± 1.45 μM	Affinity purification/ IMAM @ mPEG	[10]
Volutharpa ampullaceal perryi	IVTNWDDMGK/ VGPAGRRG	2.08 mM/4.66 mM	Affinity purification/ Zn-SBA-15-ACE	[12]
Wakame	AIYK/YKYY/ KFYG/YNKL	213 μM/64.2 μM/ 90.5 μM/21 μM/	Conventional approach	[28]
	KNFL	225.87 ± 2.70 µM	Affinity purification/ Fe_3_O_4_@ZIF-90-ACE	This study

**Table 2:** The summary of ACE inhibitory peptides have similar structure with purified peptides KNFL from food protein.

Source	Amino Sequence	IC_50_ (μM)	Reference
Soy	FFL	37.00	[2]
Soybean	IFL	44.67	[3]
Microalgae	AFL	63.10	[4]
Royal jelly	FNF	6.92	[5]
Garlic	NF	46.30	[47]
Soybean	LNF	511.4	[48]
Cuttlefish	KNGDGY	51.63	[49]
Wakame	KNFL	225.87	this study

**Table 3:** The Kinetics parameters of ACE-catalyzed reactions of active peptide.

Kinetics Parameters	Control	KNFL (192 µM)	KNFL (384 µM)
Km (mM/L)	1.564	2.238	2.278
Vmax (µM/L·min)	55.492	45.958	24.105

**Table 4:** The key amino acid residues in ACE.

Amino Acid	Lipophilicity Parameters	Number of Amino Acids	Combination Mode
L (Leu)	3.8	62	Covalent Binding /Physical Adsorption
K (Lys)	−3.9	30	Covalent Binding
R (Arg)	−4.5	26	Covalent Binding
S (Ser)	−0.8	33	Physical Adsorption
E (Glu)	−3.5	40	Physical Adsorption
Q (Gln)	−3.5	33	Physical Adsorption
N (Asn)	−3.5	31	Physical Adsorption
D (Asp)	−3.5	30	Physical Adsorption
H (His)	−3.2	21	Physical Adsorption

## Data Availability

Data is contained within the article or Supplementary Materials.

## Supplementary Materials

The following are available online at https://www.mdpi.com/1660-3397/19/3/177/s1, Figure S1: XRD spectrum of Fe_3_O_4_@ZIF-90 and Fe_3_O_4_@ZIF-90-ACE. Figure S2: The (a) nitrogen adsorption-desorption isotherm curve, (b) dV/dw (cm^3^/g·nm), (c) dV/dlog(w) (cm^3^/g) of Fe_3_O_4_@ZIF-90, and Fe_3_O_4_@ZIF-90-ACE. Figure S3: UV–vis spectra of the materials. Figure S4: Effects of initial concentration of protein (a), pH (b), immobilization temperature (c), and immobilization time (d), on immobilized ACE. Figure S5: Optimum reaction pH (a) and Temperature (b), Arrhenius plots to calculate activation energy (*E_a_*) (c), and the Lineweaver–Burk plot of free and immobilized ACE (d). Figure S6: Chromatographic purification on a Zorbax SB C18 column of Wakame protein hydrolysate (WPH) (<5KD). Figure S7: The co-elution RP-HPLC profile of a purified fraction (0.1 mg/mL) and a synthesized peptide (0.1 mg/mL). Separation was performed with a linear gradient of acetonitrile in water (containing 0.1% TFA) from 15% to 50% in 20 min at a flow rate of 0.5 mL/min. Figure S8: The concentration-dependency of the inhibitory activity by synthesized KNFL as well as purified one against ACE. Table S1: Textural properties of Fe_3_O_4_@ZIF-90 and Fe_3_O_4_@ZIF-90-ACE by the BJH model calculation. Table S2: *K_m_* and *V_max_* of immobilized and free ACE. Table S3: Purification of Angiotensin I-converting enzyme from pig lung. Table S4: Variance analysis of reaction rate-substrate concentration fitting using nonlinear regression analysis.
