# Supplementary material for: Affinity Purification of Angiotensin Converting Enzyme Inhibitory Peptides from Wakame (Undaria Pinnatifida) Using Immobilized ACE on Magnetic Metal Organic Frameworks
Source: Mar Drugs. 2021 Mar 23;19(3):177. doi: 10.3390/md19030177 (PMC8004985; doi:10.3390/md19030177)
Supplement: Supplementary file 1 [file marinedrugs-19-00177-s001.pdf]

Supporting Information for

Affinity Purification of Angiotensin Converting Enzyme Inhibitory Peptides from Wakame (Undaria pinnatifida) Using Immobilized ACE on Magnetic Metal Organic Frameworks

Xuezhen Feng [1,2], Dankui Liao [1,*], Lixia Sun [1], Shanguang Wu [2], Ping Lan [3], Zefen Wang [1], Chunzhi Li [1], Qian Zhou [1], Yuan Lu [2] and Xiongdiao Lan [3,*]

1 Guangxi Key Laboratory of Petrochemical Resource Processing and Process Intensification Technology, School of Chemistry and Chemical Engineering, Guangxi University, Nanning, 530004, China. fengxuezhenbest@163.com (X.F.), binglin0628@163.com (L.S.), wangzefen@126.com (Z.W.), 1814304019@st.gxu.edu.cn (C.L.), 15532370620@163.com (Q.Z.)

2 Medical College, Guangxi University of Science and Technology, Liuzhou, Guangxi, 545006, China. wsg_gxust1974@163.com (S.W.), luyuan0606@163.com (Y.L.)

3 Guangxi Key Laboratory of Polysaccharide Materials and Modifications, School of Chemistry and Chemical Engineering, Guangxi University for Nationalities, Nanning, 530008, China. gxlanping@163.com

* Correspondence: liaodankuigx@163.com（D.L.); lanxiongdiao@163.com (X.L.); Tel./Fax: +86-0771-3272702 (D.L.); +86-0771-3272702 (X.L.)

Table captions:

Table S1: Textural properties of Fe_3O_4@ZIF-90 and Fe_3O_4@ZIF-90-ACE by the BJH model calculation.;
Table S2: K_m and V_{max} of immobilized and free ACE.
Table S3: Purification of Angiotensin I-converting enzyme from pig lung.
Table S4: Variance analysis of reaction rate-substrate concentration fitting using nonlinear regression analysis.

Figure captions:

Figure S1: XRD spectrum of Fe_3O_4@ZIF-90 and Fe_3O_4@ZIF-90-ACE.;
Figure S2: The (a) nitrogen adsorption-desorption isotherm curve, (b) dV/dw (cm^3/g·nm), (c) dV/dlog(w) (cm^3/g) of Fe_3O_4@ZIF-90 and Fe_3O_4@ZIF-90-ACE.;
Figure S3: UV–vis spectra of the materials.;
Figure S4: Effects of initial concentration of protein (a), pH (b), immobilization temperature(c), and immobilization time (d), on immobilized ACE.;
Figure S5: Optimum reaction pH (a) and Temperature (b), (c) Arrhenius plots to calculate activation energy (E_a) and (d) the Lineweaver-Burk plot of free and immobilized ACE.;
Figure S6: Chromatographic purification on a Zorbax SB C18 column of Wakame protein hydrolysate (WPH) (<5KD).;
Figure S7: The co-elution RP-HPLC profile of a purified fraction (0.1mg/mL) and a synthesized peptide (0.1mg/mL). Separation was performed with a linear gradient of acetonitrile in water (containing 0.1% TFA) from 15% to 50% in 20 min at a flow rate of 0.5 mL/min.
Figure S8: The concentration-dependency of the inhibitory activity by synthesized KNFL as well as purified one against ACE.

Table S1. Textural properties of Fe_3O_4@ZIF-90 and Fe_3O_4@ZIF-90-ACE by the BJH model calculation.

Samples	BET surface area (m^2 g^{-1})	Langmuir surface area (m^2 g^{-1})	Pore volume (cm^3g^{-1})	Pore diameter (nm)
Fe_3O_4@ZIF-90	106.97	148.68	0.12	13.37
Fe_3O_4@ZIF-90-ACE	51.87	73.04	0.09	12.35

Table S2. K_m and V_{max} of immobilized and free ACE.

-	Kinetic Equation	R^2	K_m/mmol·L^{-1}	V_{max}/ mmol·min^{-1}	catalytic efficiency
Free ACE	y=0.0406x+0.0212	0.9892	1.962	46.296	23.596
Immobilized ACE	y=0.0486x+0.0212	0.9968	3.953	81.321	20.574

Table S3. Purification of Angiotensin I-converting enzyme from pig lung.

Purification step	Total activity（U）	Enzyme recovery	Specific activity（U/mg）	Purification fold
Homogenate	112.05	100.00	0.0021	1.0
$(NH_4)_2SO_4$ extract	75.13	67.05	0.0053	2.5
Dialysis solution	45.67	40.67	0.0102	4.9

Table S4. Variance analysis of reaction rate-substrate concentration fitting using nonlinear regression analysis.

**Items**	**Control**	**KNFL (192 μM)**	**KNFL (384 μM)**
R squared	0.989	0.986	0.989
Sum of Squares	3008.161	1457.652	393.342
Mean Squares	1504.08	728.826	196.671
df	2	2	2
P value	<0.01	<0.01	<0.01

Figure S1

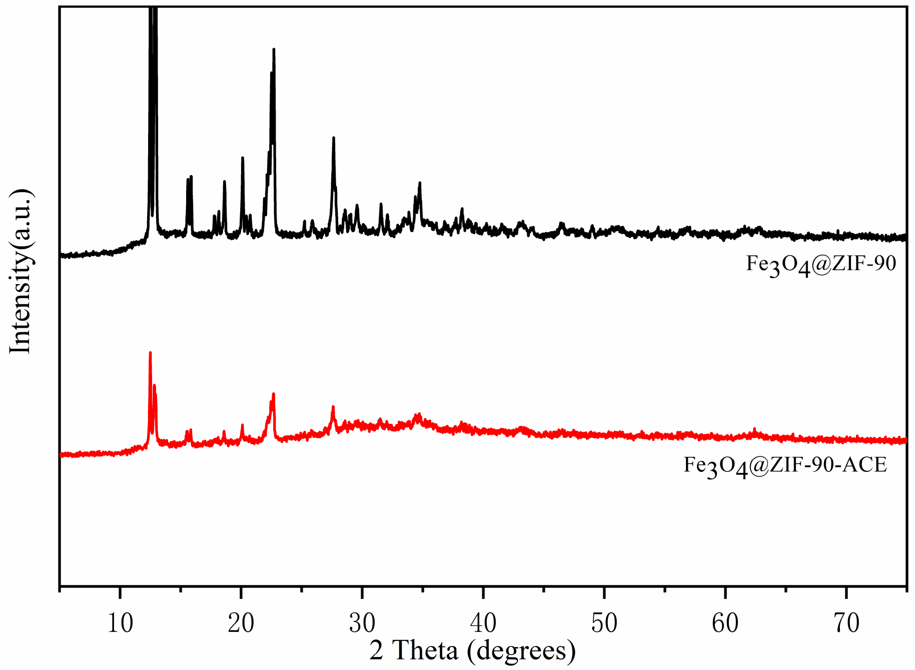

Figure 1. XRD spectrum of Fe_3O_4@ZIF-90 and Fe_3O_4@ZIF-90-ACE by X-ray diffractometer (SmartLab, Rigaku Corporation, Japan).

Four distinct characteristic diffraction peaks at 2θ=35.6°,53.8°,57.6°and 62.8°of the Fe_3O_4@ZIF-90 matched well the structure of Fe_3O_4NPs (JCPDS No.19-0629).[1] The peaks of ZIF-90 at 7.39°, 10.36°, 12.79°,14.71°, 16.45°, 18.01°, 22.19°, 24.64° and 26.72° were also observed on the Fe_3O_4@ZIF-90.[2] Howerver, the Fe_3O_4@ZIF-90-ACE had weak characteristic diffraction peaks of Fe_3O_4NPs at 30.6°, 53.8° and 62.8°. It was probably because that the impure protein of crude enzyme sample was absorbed on the surface of the Fe_3O_4@ZIF-90, hiding the peaks of Fe_3O_4NPs. Moreover, there was a slight change on the angles and peak strength, showing that the crystallinity of material were decreased on account of immobilized ACE.

Figure S2

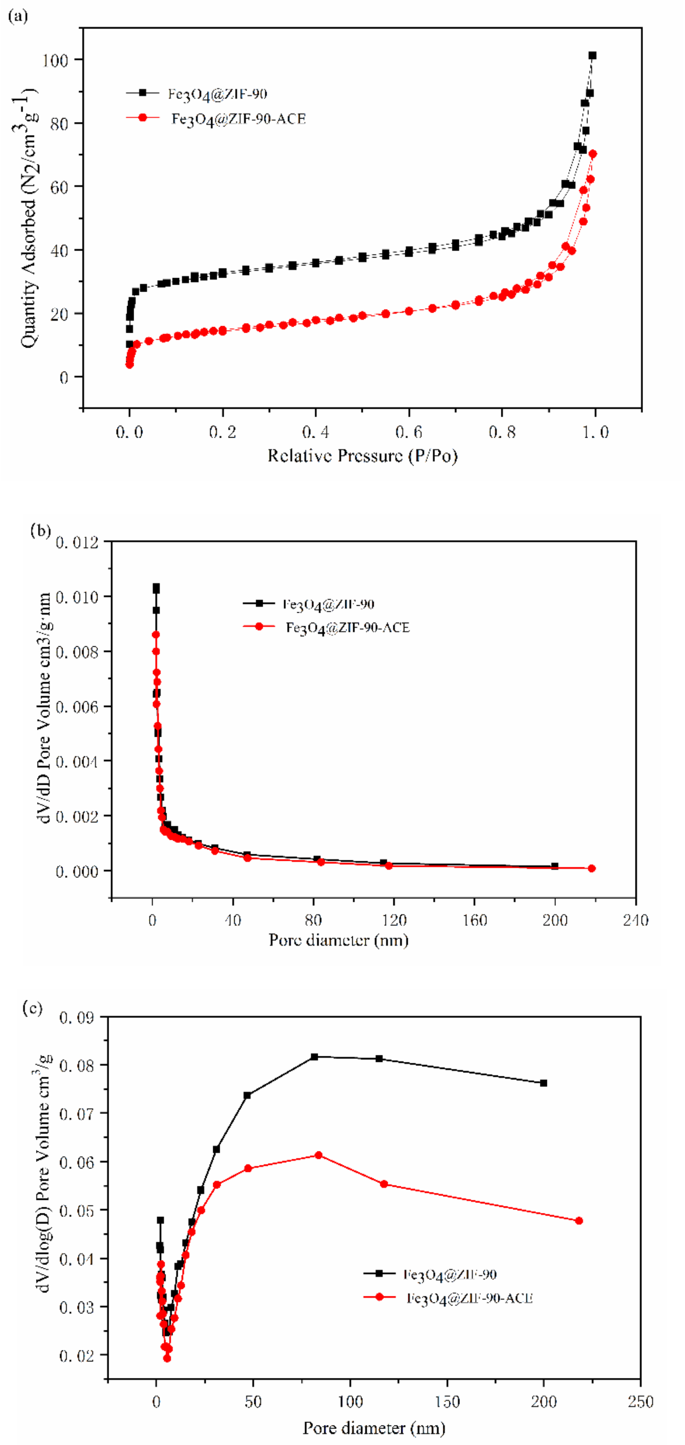

Figure 2. (a) N_2 adsorption isotherms, (b) dV/dw (cm^3/g·nm), (c) dV/dlog(w) (cm^3/g) of Fe_3O_4@ZIF-90 and Fe_3O_4@ZIF-90-ACE using a Physical Absorption analyzer (ASAP2420, Micromeritics Co., Ltd., USA).

As shown in Fig.S2, it was found that the adorption–desorption isotherms was close to type-III isotherm according to the IPUAC classification. The significant decrease in the surface area and pore size was due to the crude ACE modification which fill the pore and restrict N_2 molecules from fully accessing the pores. The similar phenomenon was also reported in the post synthesis modification of ZIF-90.[3,4]

Figure S3

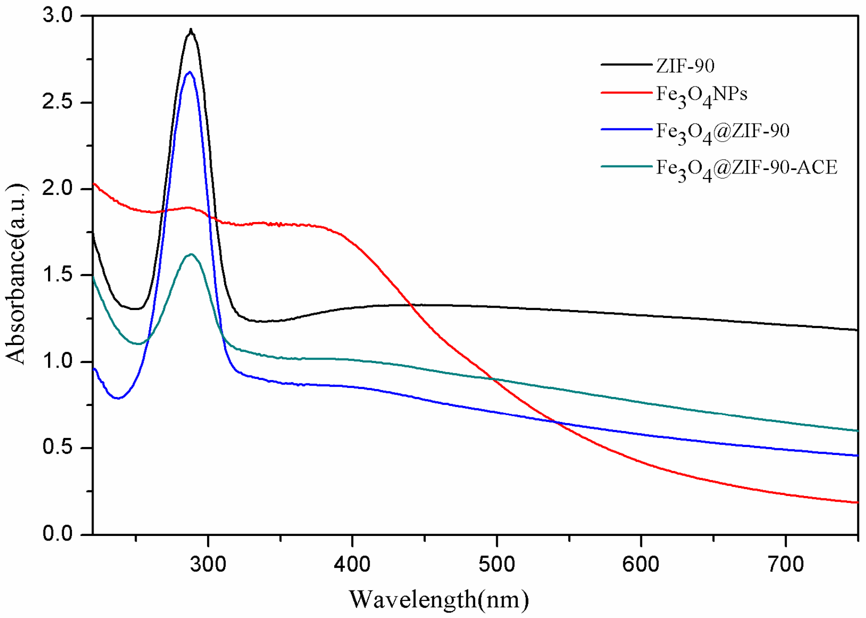

Figure 3. UV–vis spectra of the materials by UV-2550.

As shown in Fig. S3, An UV absorption peak of ZIF-90 was observed on 287 nm and Fe_3O_4@ZIF-90 and M-ZIF-90-ACE all showed similar peaks. In contrast, there was no obvious UV absorption peak of Fe_3O_4 NPs was observed for Fe_3O_4 @ZIF-90 and Fe_3O_4@ZIF-90-ACE, indicating that the absorption peak of Fe_3O_4 was mainly suppressed by the framework of ZIF-90. From the UV–vis spectra, the Fe_3O_4 NPs was successfully encapsulated into the framework of ZIF-90.[5]

Figure S4

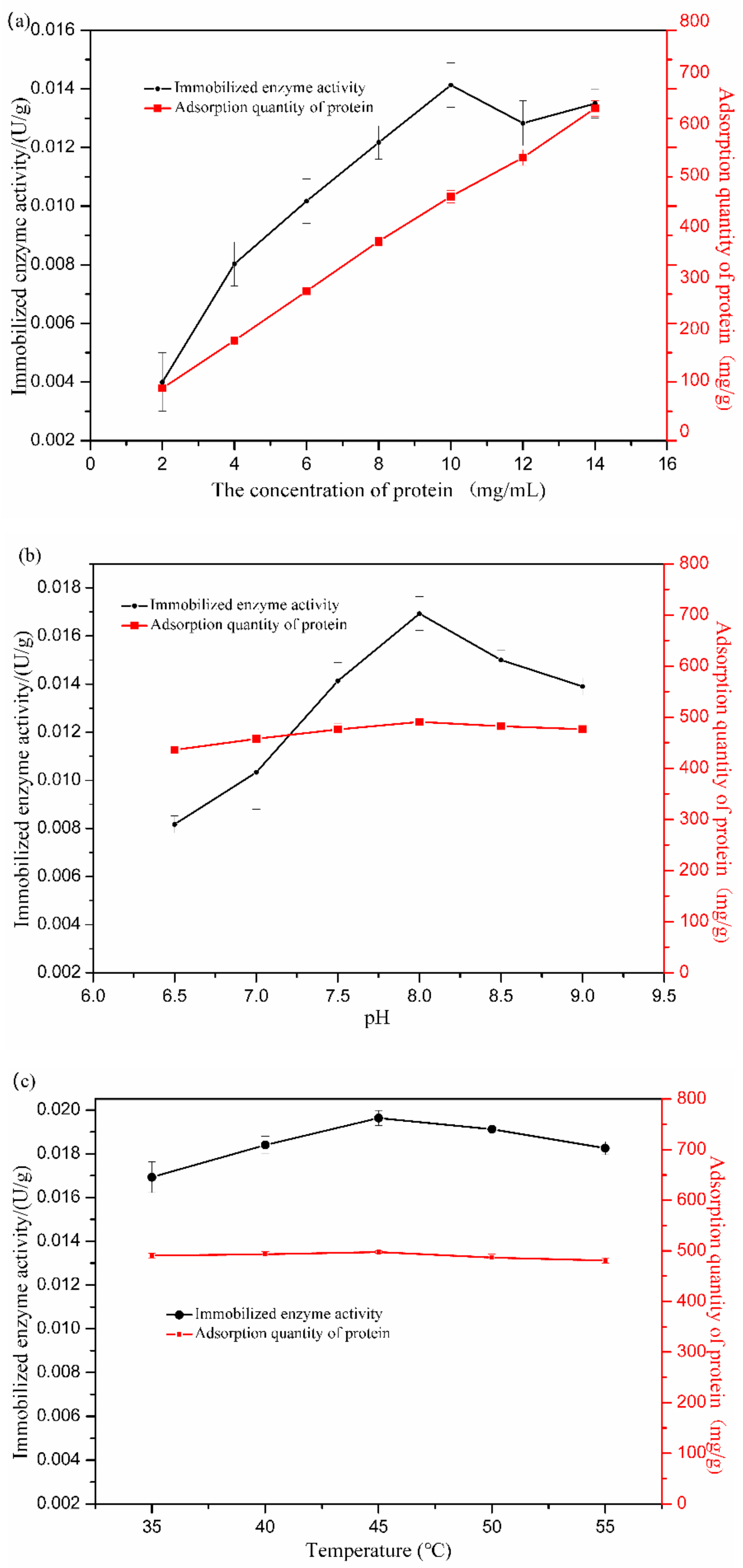
(a)
Immobilized enzyme activity
Adsorption quantity of protein
Immobilized enzyme activity/(U/g)
Adsorption quantity of protein (mg/g)
The concentration of protein (mg/mL)
(b)
Immobilized enzyme activity
Adsorption quantity of protein
Immobilized enzyme activity/(U/g)
Adsorption quantity of protein (mg/g)
pH
(c)
Immobilized enzyme activity
Adsorption quantity of protein
Immobilized enzyme activity/(U/g)
Adsorption quantity of protein (mg/g)
Temperature (°C)

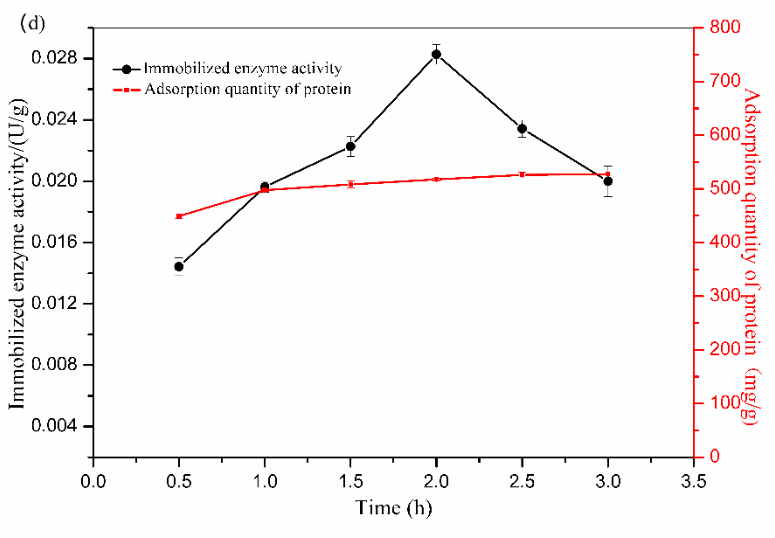

Figure 4. Effects of initial concentration of protein(a), pH (b), immobilization temperature (c) and immobilization time (d) on immobilized ACE.

ACE was immobilized onto Fe_3O_4@ZIF-90 nanoparticles through the Schiff base reaction.[6] The optimum immobilization conditions were as follow: the protein concentration of ACE crude sample was 10.0 mg/mL, pH 8.0, temperature 45℃ and the immobilization time was 2.0 h.

Figure S5

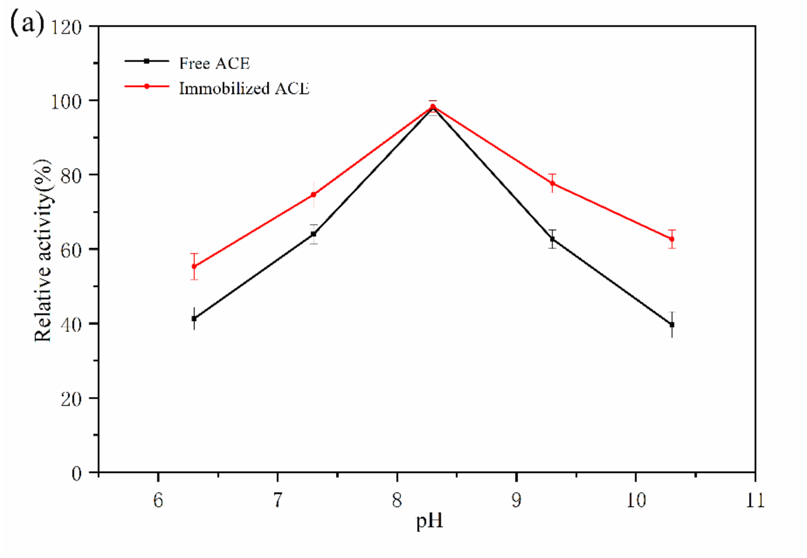
(a)
Free ACE
Immobilized ACE
Relative activity(%)
pH
0
20
40
60
80
100
120
6
7
8
9
10
11

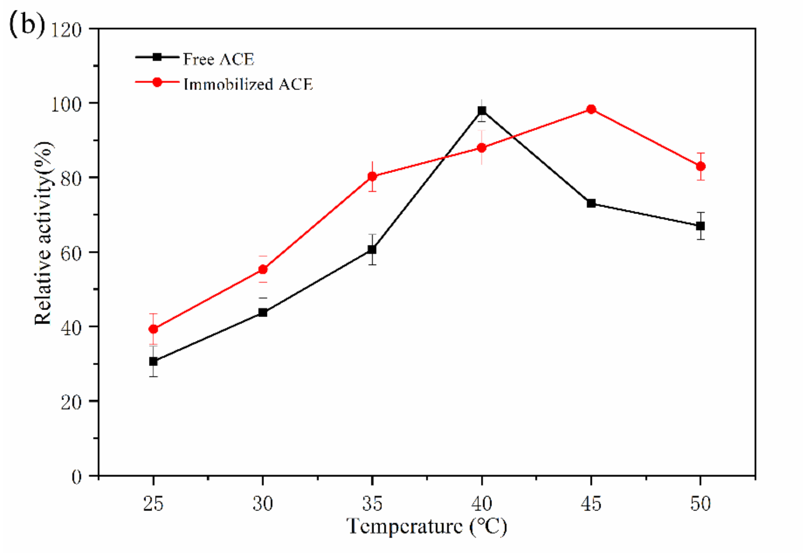
(b)
Free ACE
Immobilized ACE
Relative activity(%)
Temperature (℃)
0
20
40
60
80
100
120
25
30
35
40
45
50

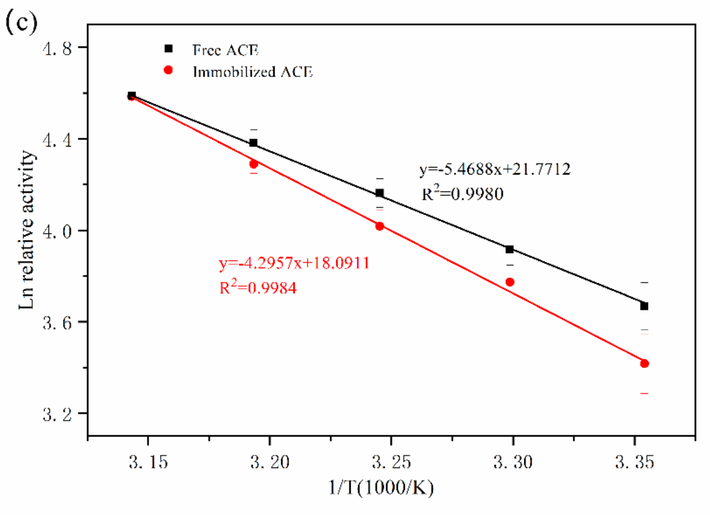
(c)
Free ACE
Immobilized ACE
y=-5.4688x+21.7712
R2=0.9980
y=-4.2957x+18.0911
R2=0.9984
Ln relative activity
1/T(1000/K)
3. 2
3. 6
4. 0
4. 4
4. 8
3. 15
3. 20
3. 25
3. 30
3. 35

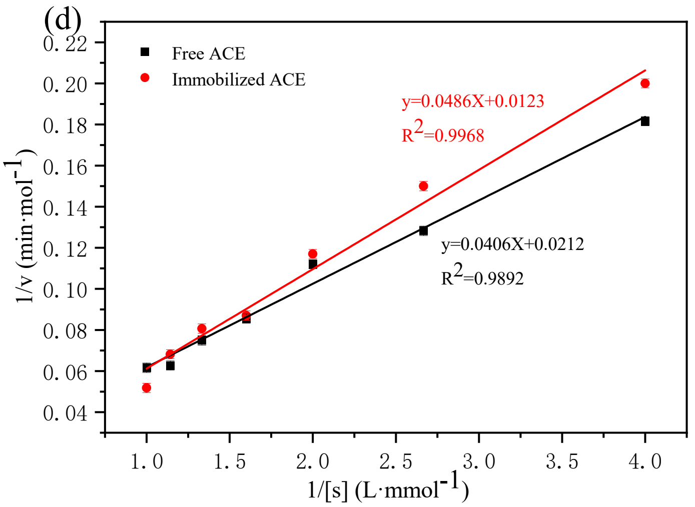

Figure 5. Optimum reaction pH (a) and Temperature (b), (c) Arrhenius plots to calculate activation energy (E_a) and (d) the Lineweaver-Burk plot of free and immobilized ACE.

Optimum pH was determined to be 8.3 for both free and immobilized ACE, indicating that the immobilization procedure did not cause any change to the optimum pH of free ACE (Fig. S5 (a)).[7] The immobilization of ACE on Fe_3O_4@ZIF-90 increased the optimum temperature to 45°C (Fig. S5 (b)) as well as the range (35–50°C) of temperatures with relative activity >80%. While the optimum temperature of free ACE was 40°C, and >40% loss of relative activity was observed between 35°C and 50°C, it was shown that the immobilized ACE enhanced temperature resistance, possibly by increasing the stiffness of the protein structure, and preventing the unfolding of ACE at high temperatures.[7]

The linear plots of ln [relative activity] vs 1/T indicates that the reactions follow first order kinetics (Fig. S5 (c)). Furthermore, the *Ea* for the enzymatic reaction with the immobilized ACE was calculated to be 45.46 kJ/mol using the Arrhenius equation, while the Ea of free ACE was determined to be 35.71 kJ/mol. This demonstrates that a higher activation energy is required for the immobilized enzyme to form the enzyme-substrate complex compared with the free enzyme.[8]

As shown in Fig. S5 (d), the K_m of the immobilized ACE (3.953 mmol/L) was 2.0-fold higher than the K_m value of the free ACE (1.962 mmol/L) (Table 2), indicating a lower affinity of ACE on Fe_3O_4@ZIF-90 towards the substrate. Immobilization usually causes a rise in K_m due to partial dissociation of ACE subunits by covalent interaction, and steric hindrance of the active site by the support or due to diffusional limitation of the substrate.[9]

Figure S6

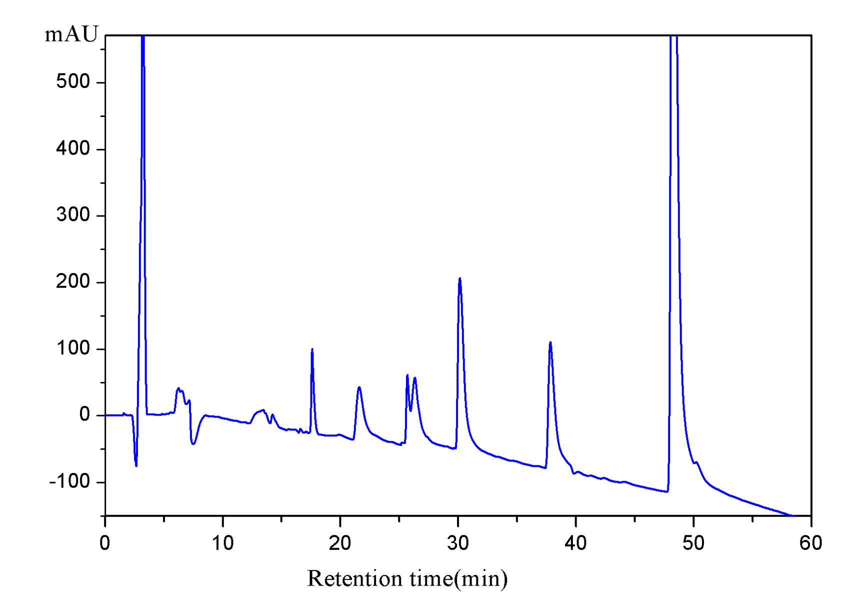

Figure 6. Chromatographic purification on a Zorbax SB C18 column of Wakame protein hydrolysate (WPH) (<5KD). Separation was performed with a linear gradient of acetonitrile in water containing 0.1% TFA (0–100% in 60min) at a flow rate of 1 mL/min.

Figure S7

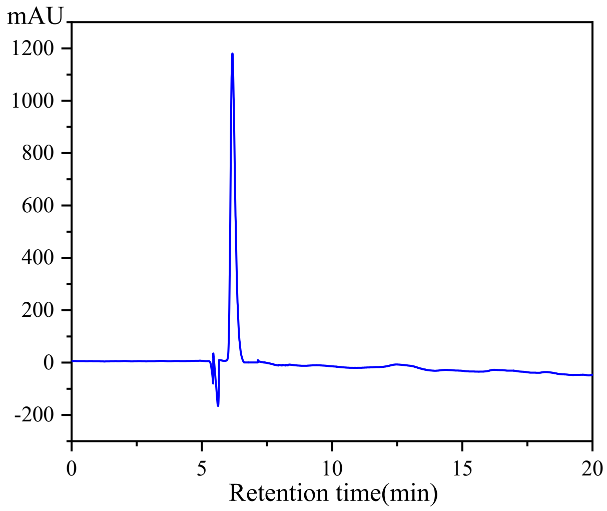

Figure 7. The co-elution RP-HPLC profile of a purified fraction (0.1mg/mL) and a synthesized peptide (0.1mg/mL). Separation was performed with a linear gradient of acetonitrile in water (containing 0.1% TFA) from 15% to 50% in 20 min at a flow rate of 0.5 mL/min.

Figure S8

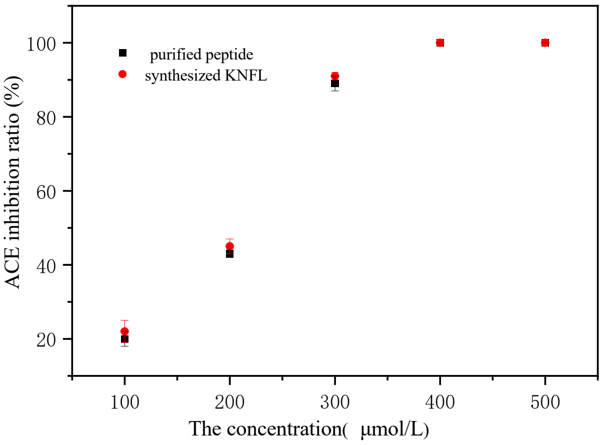

Figure 8. The concentration-dependency of the inhibitory activity by synthesized KNFL as well as purified one against ACE.

References

1. Cao, S.L.; Xu, H.; Lai, L.H.; *et al*. Magnetic ZIF-8/cellulose/Fe_3O_4 nanocomposite: preparation, characterization, and enzyme immobilization. *Bioresour. Bioprocess*.2017, 4(1):56-63.

2. Zhang, F.M.; Dong, H.; Zhang, X.; Sun, X.J.; *et al*. Post synthetic Modification of ZIF-90 for Potential Targeted Codelivery of Two Anticancer Drugs. *ACS Appl. Mater. Interfaces.* 2017, 9, 27332–27337.

3. Morris, W.; Doonan, C.J.; Furukawa, H.; *et al*. Crystals as molecules: postsynthesis covalent functionalization of zeolitic imidazolate frameworks. *J. Am. Chem. Soc.*2008,130(38),12626–12627.

4. Bhattacharjee, S.; Lee, Y.R.; Ahn, W. S.. Post-synthesis functionalization of a zeolitic imidazolate structure ZIF-90: a study on removal of Hg(II) from water and epoxidation of alkenes. *Cryst Eng Comm*.2015, 17(12):2575-2582.

5. Nosike, E.I.; Jiang, Z.; Miao, L.; *et al*. A novel hybrid nanoadsorbent for effective Hg2+ adsorption based on zeolitic imidazolate framework (ZIF-90) assembled onto poly acrylic acid capped Fe_3O_4 nanoparticles and cysteine. *J Hazard Mater*. 2020 ,392,122288.

6. Megías, C.; Pedroche, J.; Yust, M. M.; *et al*. Immobilization of Angiotensin-Converting Enzyme on Glyoxyl-Agarose. *J. Agric. Food Chem*.2006, 54(13):4641-4645.

7. Abdulla, R.; Ravindra, P.. Characterization of cross linked Burkholderia cepacia lipase in alginate and κ-carrageenan hybrid matrix. *Journal of the Taiwan Institute of Chemical Engineers*, 2013, 44(4):545-551.

8. Karam, E.A.; Abdel, W.W.A.; Saleh, S.A.A.; *et al*. Production, immobilization and thermodynamic studies of free and immobilized Aspergillus awamori amylase. *Int J Biol Macromol*. 2017,102,694-703.

9. Vineh, M.B.; Saboury, A.A.; Poostchi, A.A.; Ghasemi, A.. Biodegradation of phenol and dyes with horseradish peroxidase covalently immobilized on functionalized RGO-SiO_2 nanocomposite. *Int J Biol Macromol*. 2020,164,4403-4414.
